# Psychology as a Science of Subject and Comportment, beyond the Mind and Behavior

**DOI:** 10.1007/s12124-017-9408-4

**Published:** 2017-10-24

**Authors:** Marino Pérez-Álvarez

**Affiliations:** Universidad de Oviedo, Oviedo, Spain

**Keywords:** Behavioral sciences, Comportment, Dualistic ontology, Qualitative inquiry, Positivist scientific method

## Abstract

The turn of qualitative inquiry suggests a more open, plural conception of psychology than just the science of the mind and behavior as it is most commonly defined. Historical, ontological and epistemological binding of this conception of psychology to the positivist method of natural science may have exhausted its possibilities, and after having contributed to its prestige as a science, has now become an obstacle. It is proposed that psychology be reconceived as a science of subject and comportment in the framework of a contextual hermeneutic, social, human behavioral science. Thus, without rejecting quantitative inquiry, psychology recovers territory left aside like introspection and pre-reflective self-awareness, and reconnects with traditions marginalized from the main stream. From this perspective psychology might also recover its credibility as a human science in view of current skepticism.

A certain spirit of the times seems to require a review of psychology as a science of the mind and behavior as most established definition (American Psychological Association 2016; Gerrig 2014; Schacter et al. 2015; Stanovich 2012). Two important movements have arisen independently, one in the heart of the American Psychological Association itself with Division 5 recently renamed Quantitative and Qualitative Methods, including the Society for Qualitative Inquiry in Psychology and the Journal of Qualitative Psychology (Gergen et al. 2015) and the other in the European tradition with *The Yokohama Manifesto* for a Psychology as the Science of Human *Being* (Valsiner et al. 2016). Both assume an open pluralistic turn toward the Psyche as “complex, subjective, meaningful, and mysterious” (Valsiner et al. 2016, p. v) and as Gergen et al. say, “no practice of inquiry is ruled out a priori; multiple goals of inquiry are rendered plausible and multiple pathways may claim a situated legitimacy” (Gergen et al. 2015, p. 2).

The problem with the standard conception of psychology as a science of behavior and mental processes is that it has ended up by establishing dualistic ontology and reasoning in the psychological science with feedback from the positivist natural scientific method (Packer 2011; Slife et al. 2012). The problem with dualism, as could be anticipated, is its two-substance doctrine, *res cogitans/res extensa* (mental/physical), referring nevertheless to a *pluralistic,* non-monist *universe,* (James 1909/1977), as argued in this article. This *traditional* dualistic ontology is based on and at the same time receives feedback from an epistemology in itself dualistic in the form of the *natural* scientific method (today in crisis) with its new subject/object, theory/method and fact/value dualities, as discussed further below.

This conception obviously has its history and *raison d'être*. Not in vain, the foundation of psychology as a scientific discipline in the late nineteenth century derives from philosophical psychology studied following the positivist method of natural science in conformance with the spirit of the times (Danziger 1990; Hatfield 2002). As Hatfield says, “The new science that took hold and developed in the late nineteenth and early twentieth centuries was a transformation of the old science (or old ‘natural philosophy’)” (Hatfield 2002, p. 212). The best known, *signet* scientific movements of twentieth century psychology, such as behaviorism and cognitivism, at the bottom of their current conception are along this line.

Adoption of the natural scientific method contributed to the prestige of psychology as a science. However, psychology as a natural science for the sake of the positivist scientific method, which although up to now has represented progress, may have become a limitation if not an obstacle. One obstacle is already the implicit dual reasoning that may be influencing psychological research, as much as it believes itself to be well-fortified by the natural scientific method. But the natural scientific method adopted by the mainstream psychology (Toomela 2007), far from being exempt and in itself a guarantee of better science, structures our way of understanding and studying psychological subjects, for example, in a more analytical than holistic manner, mechanist than contextual, causal than mutualist, etc. (Slife et al. 2012). The scientific method itself may constitute an “epistemological obstacle” in the sense of the French philosopher Gaston Bachelard, as a trap for scientific knowledge and concepts set by ‘thought-habits’ (Bachelard 1938/2002), in this case derived from routine teaching of the “method” as “something in itself” (Costa and Shimp 2011; Machado and Silva 2007).

Historically, the triumphant positivist scientific method marginalized an entire rich holistic tradition which is now being missed (Diriwächter and Valsiner 2008; Toomela 2007). This resurgence of qualitative inquiry of the human being as a whole does not respond to a supposed psychoanalytical law of “return of the repressed”, but to the limitation of the scientific method itself. Labelling the natural scientific method as a source of dualism (instead of overcoming it) in no way means rejecting the statute of psychology as science. To begin with, there is no science without method. What there is not is *the* scientific method for making science (Chalmers 2013).

The question is whether the natural scientific method is the best for psychology. The assumption of psychology as a natural science may be at the bottom of its credibility crisis as a science (Ferguson 2015; Lilienfeld 2012) as well as the problem of globalization of *indigenous* western psychology as universal (Christopher et al. 2014; Henrich et al. 2010; Watters 2010). While the credibility crisis emphasizes low replication of scientific findings of hardly 40% (Open Science Collaboration 2015) in contrast to a *suspiciously* high confirmation of the hypotheses of psychological research on the order of 93% (Fanelli 2010), globalization of psychology reveals that subjects on whom psychological knowledge has been established are 96% members of Western societies (12% of the world population), a “weird” subject (WEIRD: Western, Educated, Industrialized, Rich, Democratic; Henrich et al. 2010).

The purpose of this article is to pose a relatively new conception of psychology beyond mind and behavior, in terms of subject and comportment. The argument is developed in four parts. First, the ontological and epistemological dualism of the metaphysics implicit in psychology as a science of the mind and behavior is emphasized. Second, the framework of a pluralistic ontology is presented as an alternative. Third, psychology is presented as a science of subject and comportment. The notions of subject with all its subjectivity and comportment as different from behavior are taken in the perspective of phenomenology according to its recent developments. Finally, the scope of the proposal is discussed.

## The Metaphysics of Psychology as Science of Mind and Behavior

This refers to the metaphysics implicit in ontological and epistemological philosophical assumptions which still rule in psychology. Adoption of the positivist scientific method of the natural sciences did not free it from philosophical problems, as already warned by Wundt (Lamiell 2013). The supposed aseptic methods of empirical research as the typical distinction between “dependent / independent variables” have their implicit metaphysics beginning with the axiomatic acceptance of linear causality (Valsiner and Brinkmann 2016). "There is no escape from philosophy—Jaspers says—. The question is only whether [a philosophy] is good or bad, muddled or clear. Anyone who rejects philosophy is himself unconsciously practicing a philosophy" (Jaspers 1954/2003, p. 12).

### Dualistic Ontology: Mental Processes and Behavior as Inner/Outer Dichotomy

Even when a duality does not imply dualism, in the case of the mind and behavior in the definition of psychology, it entails traditional dualism no matter how much it is dressed in new terms and metaphors. The Cartesian dualism does not find better version than in “the view of mind like software running on a physical hardware” (Everett 2016, p. 37). The definition of psychology as the “scientific study of the behavior of individuals and their mental processes” (American Psychological Association 2016; Gerrig 2014, p. 2) means at least an inner-outer dichotomy. While behavior refers to observable, factual activities in the outside world, mental processes refer to cognitive or neurocognitive activities that occur in the subject’s interior, in his mind, cognitive system or brain (Schacter et al. 2015; Westerman and Steen 2007).

It might be said that mental processes associated with neural processes may be observable depending on whether evermore refined technologies are available. But this would only pose new questions, beginning with the problem of their connection, which Descartes believed to already have resolved with the pineal gland. Turning psychological realities into physicochemical realities would also be a problem of reductionism and the consequent *mereological fallacy* of explaining what the whole does through one of its parts (Bennett and Hacker 2003) or the double-subject fallacy “me” and “my brain” (Mudrik and Maoz 2014). Even if behavior is an indispensable reference, a basic dualism still persists between two *substances*: a mental, incorporeal, unobservable, hypothetical, latent and another behavioral, material, topographical, observable. The most conspicuous example of mental *substance* today consists of the so-called cognitive processes as information processing.

Cognitive processes are mental activities postulated by cognitive psychology as top-down and bottom-up, cognitive routines, change-of-task modules, and many others, some of which are already popular, such as central executive and working memory. This renewed conception of the mind does not avoid old well-known problems such as the argument of the homunculus which consists in attributing to a sort of “inner man” what in reality makes the individual as a whole, categorical error by which an activity which is characteristically behavioral is explained by categories of a different order, for example computational or neurophysiological or the ghost in the machine as if a “cognitive demon” working on the inside produced results on the outside (Bennett and Hacker 2003; Holt 2001; Mudrik and Maoz 2014; Logan and Bundesen 2004; Weger and Wagemann 2015a). All these first-cousin problems come to a head in the basic problem: revived dualism. On one hand, the world would be converted into information and on the other into a mental representation. Within would be the cognitive processes or mental “activities”, and outside, the behavioral activities themselves, so that cognitive or mental processes would be duplications of the world and of the behavioral activity itself.

This mental duplication or representation, as much as it is conceived in already familiar terms of processing, computation, codification, storage, recovery, is nonetheless a version of the mind in a tradition that goes from Descartes to Kant. The mind as the construction of representations of the world according to the conception due to Kant has become common sense and the vision dominant in academic psychology (Packer 2011, p. 143). However, a representational approach is not inevitable. A long tradition from Vygotsky and Gestalt psychology to the ecological psychology of Gibson shows a whole variety of holistic approaches beyond the interior-exterior dichotomy (Westerman and Steen 2007). In the mental or cognitive terms themselves, non-representational alternatives may be found in terms of situated-cognition or extended mind – embodied and embedded in the world (Clark 2008; Gallagher and Crisafi 2009; Rowlands 2010; Thompson 2007).

### Dualistic Epistemology: Dichotomies of the Scientific Method

By antonomasia, the scientific method in psychology is a positivist empirical-rational method taken from the natural sciences (Danziger 1990, p. 41) and which Popper systematized as the “logic of scientific research.” The scientist, according to Popper, constructs hypotheses – or theory systems – and compares them with experience by means of observations and experiments (Popper 1934/2002). Thus the “way to think straight about psychology” follows the order: formulate hypotheses or predictions based on prior research, test, get closer to the truth and new approaches (Stanovich 2012). The problem is that the scientific method of psychology as a natural science, far from freeing it from philosophical problems, harbors an entire implicit metaphysics expressed in a number of dichotomies (Bishop 2007; Mascolo 2016; Slife et al. 2012; Valsiner and Brinkmann 2016; Yela 1987 **)**, of which three are referred to here: subject/object, theory/method and facts/values.

The subject/object dichotomy consists of a naïf realism on one hand: the properties of objects perceived as inherent to the objects themselves (Walsh et al. 2014, p. 34) and on the other, traditional mentalism: the mind as a mirror of nature (Rorty 1979). This dualism assumes that there is a reality out there to be discovered (the truth) and a method for doing so (the scientific method) so that the result is objective knowledge (accumulative, replicable). The problem is that the *objects* in psychology are interactive *subjects*, not a disinterested, indifferent reality-out there. It refers to the fundamental distinction of the philosophy of science between natural entities and interactive entities (Bishop 2007; Hacking 1999; Walsh et al. 2014, p. 474). Natural entities are fixed realities, there-given, such as electrons, chemicals, minerals, animal species or planets, indifferent to our classifications and interpretations of them. As Alan Chalmers says: “The planets do not change their motions in the light of our theories about those motions.” (Chalmers 2013, p. 137).

Interactive entities, typically human beings, however, far from being indifferent, are susceptible to influence by their classification and interpretation. This interactive nature is based on humans as interpretive and self-interpretive beings, not just scientists. The way we are in-the-world already implies some prior understanding or interpretation of the world around us (Guignon 2012; Taylor 1985). We already know about the world before discussing and theorizing about it when it appears to us as habitable, friendly, comfortable, gratifying, harsh, hostile, threatening or unsafe. The world is given us “interpreted” somehow, regardless of our awareness as interpreters. Before a child has “theory of the mind” he/she already has intersubjective, perspective, interactive and empathetic understanding (Gallagher and Zahavi 2008). More specifically, interactive entities, typically the human psychological categories, are characterized by three criteria (Walsh et al. 2014, p. 475): 1) They are socially constituted, having had no existence before social life, 2) there is a looping effect (Hacking 1995), or reactivity, which falls back on itself (e.g., the reaction of individuals to the classifications assigned to them), and 3) they are value-loaded.

The theory/method dichotomy assumes that there is a theory – constructs, hypothesis – which can be tested using an objective, independent method, which is available out there. However, it may be questioned whether this kind of independence even occurs in physics. As Nobel Prize winner Steven Weinberg says:

The interaction between theory and experiment is complicated. It is not that theories come first and then experimentalists confirm them, or that experimentalists make discoveries that are then explained by theories. Theory and experiment often go on at the same time, strongly influencing each other. (Weinberg 1995, p. 11).

In the behavioral sciences, method is theory-loaded, even if only to assume that psychological phenomena are a certain way and can be studied with a certain method (Bishop 2007, p. 50).

In fact, “scientific method” does not really exist as something in itself, *universal*. What does exist is the “scientific method discourse”. The discourse of scientific method was established to serve two purposes: Mark the limits between what is science and what is not, and give scientific knowledge status in society, particularly in research funding agencies and as reliable evidence in courts of law (Andersen and Hepburn 2015; Haack 2010). Delimitation and status are especially relevant in medicine with regard to alternative medicine and in psychology, which claims to be a natural science. Far from autonomy as something neutral, the “scientific method” is not exempt from theory: interpretations and subjective judgments. As in subjective tests, the “scientific method” is still equally subjective, as experimental researchers have already *made a judgment* at one time about what it is important to study. According to Slife and Gantt:

The only difference between so-called objective and subjective tests is the time at which subjective judgments are rendered, with judgments for objective tests rendered before the test and judgments for the subjective tests rendered after the test. This distinction is analogous to so-called objective and subjective methods of science. […] Similar to objective tests in education, experimenters have simply decided beforehand how they intend to subjectively carry out their studies and defend their findings. (Slife and Gantt 1999, p. 1459).

Its existence as “discourse” rather than true “method” can be seen in scientific publications. The typical IMRAD format (Introduction, Method, Results, Analysis, Discussion) is more a retrospective reconstruction than a reflection of research, which has no single standard model. As the 1963 Nobel Prize winner in Medicine, Peter Medawar, said in reference to biomedical research, scientific papers do not reflect how the results are produced, leading to understand (fraudulently) that they follow a hypothetical-deductive logic (Howitt and Wilson 2014; Medawar 1996). With regard to psychology, the phenomenon has been defined as HARKing (Hypothesizing After the Results are Known) and consists of (re)formulating the hypothesis after the results are known (Kerr 1998).

The facts/values dichotomy forms part of the objectivism which impregnates positivist conceptions of the social sciences, assuming that scientific knowledge is a matter of facts, not values. This dichotomy is a correlate of the subject/object dichotomy where it is assumed that there is a reality of facts out there, like the theory/method dichotomy, but in this case it assumes an objective method which deals directly with data. However, the facts themselves are not independent of theory and methods of research, beginning with the reasons such facts are relevant and merit study (Packer and Addison 1989). Facts exempt from this could only be imagined from a conception which takes itself as a natural, positivist-methodologist science. From a conception of psychology as the dialectical-contextual science of *being* human, there would be no situation indifferent to values, without ethical implications. According to Pedersen and Bang,

There is no non-existential situation; a situation in which a person takes part always has existential qualities. There is always a core dimension of importance; there is *no such thing as a neutral situation indifferent to values in a person’s life*. (Pedersen and Bang 2016, p. 473, emphasis in original).

Without denying that there are *real* facts, what is affirmed is that they are real *facts* for something and someone, not exempt or indifferent.

### Overcoming the “Psychological Complex” of the Method in Favor of Complexity of Psychology

Psychology adopted the positivist “scientific method” to enroll as a natural science. This strategy contributed to its institutional prestige, but did not free it from dualistic metaphysics as has been shown. The “scientific method” is not used in the natural sciences because in fact it does not exist as anything but the “discourse” argued in the social and health sciences. As Weinberg says with regard to physics:

We do not have a fixed scientific method to rally round and defend. I remember that I spoke years ago to a high-school teacher who explained proudly that in her classes they were trying to get away from teaching just scientific facts and instead give the students an idea of what the scientific method was. I replied that I had no idea what the scientific method was, and I thought she ought to teach her students scientific facts. […] I think it does no good for scientists to pretend that we have a clear *a priori* idea of the scientific method. (Weinberg 1995, p. 8, 10).

When “science as it is done” is studied in a biology laboratory, the research attitude does not consist of applying the “scientific method” or the findings that come from it, but in an often handcrafted practical material construction process, including operations, equipment, discussions and arguments (Latour and Woolgar 1979–1986). The testimony of James Watson in his book, *The Double Helix*, in which he tells about the discovery of the structure of DNA, shows that the process of discovery was anything but straightforward. As he says in the preface:

As I hope this book will show, science seldom proceeds in the straightforward logical manner imagined by outsiders. Instead, its steps forward (and sometimes backward) are often very human events in which personalities and cultura1 traditions play major roles. […] I believe, there remains general ignorance about how science is "done." That is not to say that all science is done in the manner described here. This is far from the case, for styles of scientific research vary almost as much as human personalities. On the other hand, I do not believe that the way DNA came out constitutes an odd exception to a scientific world complicated by the contradictory pulls of ambition and the sense of fair play. (Watson 1968, Preface).

Skinner’s case is notable in psychology. Far from being a positivist (Smith 1986), Skinner offers a *radical* criticism of the methodologism governing the psychology of the mid-twentieth century represented by *methodological* behaviorism according to his own distinction with regard to his *radical* behaviorism (Skinner 1945), when he describes his “case within the scientific method” (Skinner 1956). In his explanation of his “scientific behavior”, Skinner ridicules the scientific method typically hypothetic-deductive, when he discusses his methodological principles, “When you run onto something interesting, drop everything else and study it”, “Some ways of doing research are easier than others”, “Some people are lucky”, and “Apparatuses sometimes break down” (Skinner 1956). As he says,

I never faced a Problem which was more than the eternal problem of finding order. I never attacked a problem by constructing a Hypothesis. I never deduced Theorems or submitted them to Experimental Check. So far as I can see, I had no preconceived Model of behavior—certainly not a physiological or mentalistic one, and, I believe, not a conceptual one. (Skinner 1956, p. 227).

As you will recall, Skinner’s “radical” behaviorism means “total” in the sense in which a psychology which prides itself in being a science should not leave anything out for methodological reasons, precisely as methodological behaviorism does when it rejects private events (feelings, thoughts) as unobservable, to later reintroduce them as intermediate variables and hypothetical constructs. To Skinner, private events can and must be studied “in their own right” (not as hypotheses), because they are observable, with the particularity of being so for a single person: oneself. The question for Skinner is then to study how society (“verbal community”) manages to teach one, starting with children, to account for the subjective world (“private world”). The question lies, has its roots, another sense of radical, in the language (“verbal behavior”). The subjective world has its roots in the verbal community through language. To Skinner, development of the private world and verbal behavior are contemporary (Skinner 1945).

With the scientific method, a certain “psychological complex” in psychology might be spoken of, in which the fear of not being seen as science leads it to *fixation* with method as if that were something in itself. In fact, “scientific method” is a term that should be avoided as inaccurate and misleading (Lilienfeld et al. 2015). Instead, methodological pluralism is proposed (Slife and Gantt 1999), beginning with the framework of a pluralistic ontology.

## An Ontological Framework for Psychology as a Radically Human Science

Following this argument, certain implicit metaphysics consisting of a dualistic ontology with feedback from the natural scientific method would be at the basis of the persistent dualism in psychology. It is understood that the identification of psychology as a natural science was probably due more to its prestige than to its own complexity as implied by its study. It is time to reconceive psychology as a human science (social, cultural, behavioral), without a “complex”. In order to do so, an ontological question should be raised concerning the place of psychology in a pluralistic ontology, not dualistic or monistic.

### The Place of Psychology in a Pluralistic Ontology

The alternative to dualism is not monism, in reality a variant of dualism itself, but pluralism, as already proposed by William James in James 1909 in *A Pluralistic Universe*. James’s thesis is a defense of the pluralistic against the monistic view (James 1909/1977; Wendt and Slife 2009).

A pluralistic ontology does not reduce reality to two substances (dualism) or to one (monism). Realities have many forms: experiential (pain, feelings, thoughts), physical (electrons, atoms, cells, organisms, typewriters, planets), institutional (languages, cultures, family relationship systems, collective imaginaries, world views) and abstract (mathematics, theorems, theories, geometry). Not all of them are related to all the others, nor are some reduced to others. A toothache is as real as the typewriter out there (Skinner 1945). The distance between two bodies is not in itself corporeal. There is no way to do geometry without drawing lines, but for example, the structure of the polyhedron is not reduced to or deduced from the lines drawn. Although all reality has an aspect or physical moment (the *damaged* tooth in the case of the tooth*ache*, straight lines forming a two-dimensional polyhedron *perceived*, however, like a three-dimensional cube), matter must not be confused with physical corporeity. “Matter” here has a sense similar to *real* whether physical, experiential or conceptual. But without physical materiality of some type nothing would exist either, so materialism is privileged as philosophical doctrine instead of, for example, idealism, spiritualism or mere pragmatism. The primacy of physical materialism may be accepted without being the final word.

Materialism, in the long tradition referred to here, is the philosophical materialism developed by the Spanish philosopher Gustavo Bueno (1972, 1990, 2016). Philosophical materialism is based on an idea of matter and distinguishes three genres of materiality, as specified below.

The idea of matter is not a scientific idea, but philosophical. The fact that a science, typically neuroscience, declares itself monistically materialist (everything is physico-chemical) is not a neuroscientific finding, but a philosophical idea. The idea of ontological-general matter replaces the idea of *Being one and immaterial* of the ontological (Eleatic-Aristotelian) tradition. Contrary to the tradition, philosophical materialism denies the pure immaterial spirit. In this sense, the idea of matter is *negative* (denial of the immaterial), but *positive* in the sense of affirming an infinite plurality " for which the denomination of *ontological-general Matter* is more fitting than *Being*" (Bueno 2016, p. 45). Consequently, the idea of matter does not necessarily imply “physical matter”, but in the end, just *one* of the genres of materiality.

The notion of matter is characterized by three attributes: plurality, discontinuity and co-determination. The plurality of matter was already referred to when its heterogeneity was compared to monism. Discontinuity obeys Plato’s principle of *symploke* (*Sophist*, 251e 253e).

The idea of *symploke* by which “nothing is isolated from everything else, but not everything is connected to everything else, otherwise, nothing could be known”, is taken by Bueno as the beginning of his pluralistic ontology. This principle argues for the irreducibility between different categories of reality, even when they share elements, such as, for example, neurobiology and psychology or sociology and history. Thus, the triumphal march of Napoleon into Jena on 14 October 1806, may serve as an example. Even when certain neurophysiological and psychological conditions of Napoleon as an individual form part of his actions, neither his hormonal nor psychological state (for instance, self-esteem), explain the historical fact. Historical reasons emphasized by historians account for this rather than any physiological or psychological reason. Hegel said he saw “The World Spirit on horseback” in Napoleon’s entry. Co-determination refers to relationships of influence between parts of reality. Co-determination is at the root of the continuous evolution of the world and historical-cultural change.

Ontological plurality is organized in the philosophical tradition into three planes, kingdoms or worlds: World, Soul, God, which philosophical materialism rework respectively as genera of materiality, M_1_, M_2_ and M_3_. M_1_ refers to physical-body, from electrons to planets, M_2_ refers to the subject with its subjectivity and behavioral activity, and M_3_ refers to the objective world (abstract, such as mathematics and supraindividual such as social institutions and material culture). The “Necker cube” offers an example involving the three genres of materiality (Bueno 2016, pp. 233–244). To begin with, the ambiguous perception which the phenomenon consists of would be a clear example of M_2_: a cube *perceived*, incorporeal, three-dimensional solid. M_1_ would be the straight lines drawn on a two-dimensional plane. M_3_ would be the geometric laws that organize the structure of polyhedrons with their transformations, rotations, etc. The “Necker cube” perceived is thus, above all, an incorporeal M_2_ content, but at the same time material with M_1_ parts consisting on one hand of the many connected lines drawn in it, and on the other the neurophysiological correlates involved. Similarly, within its subjectivity as a perceptual phenomenon, it is still objective (M_3_), as the same figure (and no other) can be imposed at any given moment, for example, on a group of individuals in an experimental session. However, the “Necker cube” as psychological reality (M_2_) not only is not reduced to, nor deduces from M_1_ or M_2_, but constitutes the phenomenon that it is.

The doctrine of the three genres of materiality has similarities with tripartite ontologies occurring throughout the twentieth century, especially, Simmel, Popper and Penrose. The purpose of a tripartite ontology comes from the need for a third genre or *world* along with the two most obvious of subject and object (soul, world). The role represented by God in traditional metaphysics having fallen, its position now falls back on the postulation of abstract, supraindividual realities, immanent to the world.

Thus, George Simmel in his 1910 work *Hauptprobleme der Philosophie* (*Main problems of philosophy*) needs to recur to a “third kingdom” of ideal contents (objective, suprapersonal) to understand and sustain a nonreductive relationship between subject and object (Simmel 2006). Karl Popper introduces World 3 as the third world relatively autonomous from the other two worlds, physical (World 1) and mental (World 2) with regard to the mind-body problem (Popper and Eccles 1977). World 3 is also in coherence with Popper’s own predisposition “to objectivity, and to individuals’ engaging, in their work, with something beyond themselves, and thereby transcending themselves, whether in art or science or thought.” (Boyd 2016, p. 17). Roger Penrose, also discussed three worlds: the Platonic world of mathematical forms, the physical world and the mental world (Penrose 1994).

A tripartite ontology, compared to a dualistic one, is necessary for three reasons. In the first place, because of the problematic dichotomies of dualism referred to above. In the second, because of reductionist monism (typically physicalist) to which dualism seems headed toward when a pluralistic ontology is not assumed, and in the third place, because of the abstract objective statute of scientific knowledge (M_3_). The third genre, M_3_, also represents culture as a supraindividual institutional reality, considered from this perspective as a condition of possibility of psychological realities themselves.

Philosophical materialism opposes all reification or hypostasis, as well as all reduction, in favor of dialectical co-determining relationships between the various materialities, not mere interactions (Bueno 1972). Co-determination with regard to the notion of catalysis refers to a relationship of mutual reciprocity given propitious contexts (catalysts), such as for example, the discriminative stimulus in relation to operant behavior (see below). Co-determination is offered as an alternative to linear causality. The place of psychology (“World 2”, M_2_) is *in media res* of the physical-corporal (“World 1”, M_1_) and supraindividual institutional (“World 3”, M_3_) realities. Psychology, as subjective-behavioral material, far from being reducible on one hand to biophysical or on the other to cultural or “objective spirit”, participates in both realities and mediates between them. Not in vain is psychology characterized as a *liminal* science (Valisner 2013, p. 137; Valsiner 2014b, p. 6). It is important to recognize the liminal character of psychology with a view to perceiving its mediating role as a science of intentional interactive processes.

Psychological materiality would thus have an inter-mediator role, configuring the human world. This refers above all to a behavioral mediation characteristic of an operant subject whose “operant behaviors” are understood as inherently intentional and significant. Behavioral mediation is pointed out to emphasize the practical-effective instrumental aspect of human action (versus “mental”). This mediation, put forward by Popper, may be identified as “bio-psychological” (Doria 2012). “Semiotic-material mediation” would be equally conceivable (Doria 2012).

The idea of mediation – behavioral, biopsychological, semiotic – implies a dialectic, two-way and mutually constitutive relationship between culture (World 3, M_3_) and the subject (mental world, M_2_), including the corporal subject (M_1_). In a conventional manner (deceptive), it might be said that culture is inscribed on the mind and brain and in turn, the mind and brain act in the world. But it cannot be said that the mind or the brain act and think without incurring in the *mereological fallacy* (Bennett and Hacker 2003), which consists of attributing to a part functions which pertain to a whole, in this case, the individual, the person or the subject. Mediation assumes a holistic subject which does not carry the world *inside* (coded or represented), or act *from* inside (mind, brain), but is a subject *situated*-in-the-world. It refers to a subject constantly changing within its permanence, always *in media res*, on the border of irreversible time (Valsiner 2016).

Mediation operates on the border between the subject and the world, a boundary which is also temporal between the here-present and a co-present future, suspected and foreshadowed. Internalization finds reconsideration beyond the overused external/internal dichotomy. According to Zittoun and Gillespie:

Internalization is not putting “in” what has been “out”: first, semiotic guidance operates at the boundary of self and the world; and second, it allows guiding one’s inner flow of experience through semiotic configuration now self-initiated. […] strictly speaking, there is nothing that becomes internalized, rather, there is an external world that produces an experience. The experience is called ‘internal’ merely because it is not accessible to observers, it has private qualia that cannot be captured from an observers’ perspective. (Zittoun and Gillespie 2015, p. 484).

Psychology not only mediates, but participates constitutively in both the physical-material (physiological) and cultural and abstract (“objective spirit”) ontological genres. It refers to a three-dimensional ontology of psychological phenomena.

### Three-Dimensional Ontology of Psychological Phenomena

Given the place of psychology (M_2_) in the middle of and in relation to other realities (M_1_, M_3_) according to the ontology of three genres followed, it is worth mentioning a triple dimensionality of psychological phenomena. It is important to do this at all in order to recognize that not everything is psychological (which would be a type of reductionism), nor is what is precisely psychological separate from the physical (M_1_) or institutional (M_3_). This refers to how psychological phenomena participate more or less conspicuously or relevantly in non-psychological aspects (M_1_ and M_3_). Several trilogies suggest this triple dimensionality in their own way. They are found in Ortega y Gasset as *vitality, soul and spirit* (Ortega y Gasset 1924/1966), in Merleau-Ponty as *physical, vital (virtual) and human order* (Merleau-Ponty 1942/1963; Thompson 2007, p. 74), in Binswanger as *umwelt* (“around world”), *eigenwelt* (“own world”) and *mitwelt* (“with world”) (Binswanger 1958; Sullivan 2015, pp. 28–31) and in Strasser as *bios, pathos* and *logos* (Strassers 1977).

Freud and Skinner also have *their* version. In Freud it would be the trilogy *id, ego, superego*. Skinner, who refers to Freud in this respect emphasizes that, “Human behavior is the joint product of 1) the contingencies of survival responsible for the natural selection of the species and 2) the contingencies of reinforcement responsible for the repertoires acquired by its members, including 3) the special contingencies maintained by an evolved social enviromental” (Skinner 1981, p. 502).

It is worth mentioning the recent recovery of this trilogy in its classic terms *body, soul and spirit* (Weger and Wagemann 2015b), along a line similar to Ortega y Gasset (1924/1966). Needless to say, the notions of soul and spirit are far from any Cartesian or spiritualist connotation. On the contrary, they agree well with the conception under discussion here, even when not thought of within the coordinates of philosophical materialism. Insofar as we are concerned, the conception of Ortega y Gasset is going to be taken up again with respect to the ontological aspects we wish to show.

Ortega y Gasset proposed making a “person’s tectonics” describing his topography in terms of *vitality*, *soul*, and *spirit*. Vitality refers to that “portion of our psyche which lives infused in the body,” the “intrabody” or lived body, with its vital force and often unclear latent reasons. The soul refers to the “region of feelings and emotions, desires, impulses and appetites.” The spirit is understanding and will, “rational operations” abiding by “norms and objective requirements.”

This tectonics of the human psyche is important for three reasons. In the first place, because it emphasizes the double bodily-vital (bios) and objective-conceptual (logos) root of the psyche (pathos). In the second place, it makes it possible to go beyond the inner-outer dichotomy and its implicit dualism by conceiving private events as nested processes that form a constituent part of our activity *in* the world. Cognitions would not be a separate reality, but partial processes that mesh with other parts of what a person is doing and with aspects of the present situation (Westerman and Steen 2007). In the third place, it provides a structure of the person on which psychological reductionism transcends. Not considering a pluralistic ontology (trigeneric) would easily lead to reductionist psychologism, and more frequent today, to cerebrocentrism, as if everything emerged from bottom up in an assumed (unexplained) growing neural complexity.

Allow me two quotes from Ortega y Gasset on the interplay of these three dimensions, how all of them are intimate, but the most personal is the experiential (soul, pathos). The other two dimensions may become impersonal either because they are objective activities that all of us would do the same because they form part of a shared normativity (spirit, logos) or else by “falling” into generic bodily processes (vitality, bios).

I think to the extent that I allow the laws of logic to be met in myself and I mold the activity of my intelligence to the being of things. Therefore, pure thought is in principle identical in all individuals. The same is true of our will. If it functioned strictly, accommodating itself to what “must be”, we would all want the same thing. Our spirit, then, does not differentiate us from others, to the point that some philosophers have suspected that there might be a single universal spirit, of which our own is only a moment or pulsation. When we think or desire, we abandon our individuality and start to participate in a universal world into which all other spirits flow and participate as ours does. So that even as the most personal part of us – if person is understood as the origin of one’s own acts – the spirit, strictly speaking, does not live on itself, but from the Truth, Law, etc., of an objective world which supports it, and from which it receives its particular contexture. In other words, the spirit does not rest on itself, but has its roots and principle in that universal, extrasubjective world. A spirit which functioned for itself and unto itself, in its own way, taste and temperament, would not be a spirit, but a soul (Ortega y Gasset 1924/1966, p. 86)

Our body, Ortega y Gasset says, does not live on itself and from itself either. Species and inheritance are extraindividual powers that act on the body of each individual. Thus individual vitality would still be a participant in a torrent of supraindividual, “cosmic” vitality to the extreme that there would be no lack of circumstances in which the body, so to speak, predominates over individuality. In this respect, Ortega y Gasset cites situations of maximum bodily exaltation, such as inebriation, orgasm and orgiastic dances, as “bringing with them the dissolution of individual awareness and a delicious annihilation in cosmic unity.” Laughter and crying could be included here as limits of human behavior where the body itself seems to take control and respond for one, according to the study by German anthropologists and philosopher Helmuth Plessner (1970).

The predominance of spirit and body tends to deindividualize us, and at the same time, suspend the life of our soul. Science and orgy empty us of emotion and desire and throw us out of the enclosure in which we all lived, confronting all others, lost in ourselves, and turns us out into extraindividual regions, whether the superiority of the Ideal, or the inferiority of the Vital and cosmic. The soul or psyche is then the center of the individual, the private enclosure against the rest of the universe, which is somehow a public region. As it does not coincide fully with either cosmic vitality or objective spirit, the soul or psyche represents individual eccentricity. We feel like individuals because of that mysterious eccentricity of our soul. Because against nature and spirit, the soul is just that: eccentric life (Ortega y Gasset 1924/1966, p. 88-90).

The essential human eccentricity – always taking position on oneself without coinciding fully with itself – is equally emphasized by Plessner’s adualistic anthropology (Plessner 1970).

## Beyond Mind and Behavior, Subject and Comportment

On the basis of a pluralistic ontology, not dualistic or monistic, but three genres of reality (physical-corporal, subjective-comportmental, objective-supraindividual), a three-dimensional ontology of psychological phenomena themselves has been emphasized. As psychological phenomena, far from being reducible, are mediators (behavioral, semiotic) between the various planes of reality, they themselves share in both “objective spirit” and physiological corporality. These psychological phenomena being located *in media res* of the rest of realities, they are not well captured following the standard conception of psychology as behavioral science and of mental processes. This conception remains prisoner of an implicit dualism due to the basic dualistic ontology fed back in turn by an equally dualistic epistemology (“scientific method”), in accordance with what was said.

In this critical appreciation, the problem is not in psychology as *science*, but what kind of science psychology *is*. The problem would be in psychology attempting to be a natural science instead of a human, cultural, social, or behavioral science. Neither is the problem the duality. A double subjective-interactive aspect is imposed on the *nature* of psychological phenomena as experiences and ways of acting. The problem would be in its delineation in inner mental and external behavioral terms, more sewn together than interwoven. In this respect, the new well-known framework of subject and comportment are proposed, instead of mind and behavior, as the duality which best captures the place and role of psychology in the whole of a “pluralistic universe”.

### The Operant Intentionality Inherent in Comportment

The notions of subject and comportment are taken up again in the phenomenological perspective (Husserl, Heidegger, Sartre, Merleau-Ponty, Ortega y Gasset). Not in vain is phenomenology a radically adualistic philosophy of great interest for the renovation of psychology (Dreyfus 1992; Gallagher and Zahavi 2008; Thompson 2007). Taking up phenomenology again in the context of the pluralistic ontology of philosophical materialism, there is no risk of subjectivism and transcendentalism, as phenomenology seems at times to fall into idealism and talk about *sub specie aeternitatis*.

While subject refers to an embodied, embedded, and enacted subject, comportment refers to a constitutive relationship of subject with the world, different from the notion of behavior as the instrument of the mind (Jacobs et al. 2014; Merleau-Ponty 1942/1963; Thompson 2007; Yela 1987). The concept of comportment introduced captures the “*unifying structure of embodied affective (and cognitive) engagement with the world*, as the most general term to refer to all-encompassing changes.” (Jacobs et al. 2014, p. 90). The subject and the world are mutually constituted in a circular structure in which each of the terms exists and is logical with regard to the other. The way we are positioned is characterized by a phenomenical *from-to structure* (perceptive and operative), from which we perceive something and we operate on it, which always presumes tacit knowledge (Polanyi 1983, p. 11). The human biophysical structure itself propels both forward and outward, opening a way for itself on a temporal and spatial horizon. Self-transcendence of the organism and immanent purposefulness of life may be spoken of as well as going beyond the given condition of oneself (Tateo 2014; Thompson 2007).

This essential way of being in the world already implies, in Husserl’s terms, an operant intentionality (*fungierende intentionalität*) followed by Merleau-Ponty (*intentionalité opérant*), before any mental or representational intentionality (Merleau-Ponty 1945/1962; Thompson 2005). According to Merleau-Ponty following Heidegger, the relationship between one and the world is not primarily a relationship of subject to object or the reverse, but a mutually constitutive relationship expressed as being-in-the-world.

The world is inseparable from the subject, but from a subject which is nothing but a projection of the world, and the subject is inseparable from the world, but from a world which the subject itself projects. (Merleau-Ponty 1945/1962, p. 430).

Belonging to the world this way means that our essential way of relating to things is not purely sensorial and reflection, nor cognitive or intellectual, but rather corporeal and practical, articulated by a “motor intentionality.” This body motor intentionality-environment constitutes what Merleau-Ponty calls the “intentional arch” which subtends our relationship with the world integrating sensitivity and motility, perception and action (Merleau-Ponty 1945/1962, p. 136). The intentional arch and being-in-the-world are not exactly subjective or objective, or mental or physical. They are existential structures previous to and more basic than those abstractions (Thompson 2005, p. 410). From this perspective, the mind ceases to be something interior and is conceived as a relationship, and the world ceases to be something exterior and is conceived as the medium.

Operant intentionality is inherent in comportment. It refers to a notion of comportment which involves implicit knowledge and understanding of the world, not a mere behavioral action, but meaningful comportment (Yela 1987). Comportment incorporates both bodily subjectivity and normative objectivity, according to the three-dimensional ontology noted above (Ortega y Gasset 1924/1966; Weger and Wagemann 2015b). It is a sense of comportment which captures the single structure of our bodily, affective and cognitive articulation with the world (Jacobs et al. 2014, p. 90). This notion of comportment (*Verhaltung*) is found in Heidegger’s *Being and Time* with reference to “know-how” having to do with concrete situations. In English it is translated as “ways of behaving/behavior”, “ways in which Dasein comports itself” (Jacobs et al. 2014, p. 108, nota 1) or “comportment” (Sembera 2007, p. 233). This notion of comportment is the same as developed by Merleau-Ponty in his Merleau-Ponty 1942 work entitled in French *La Structure du comportement*, translated into English as *The Structure of Behavior* (Merleau-Ponty 1942/1963). “Structure” here means the gestalt form or unit of sense (Thompson 2007, p. 67). Like the bridge described stone by stone or by the arch it forms, comportment defines not some stones or others (“behaviors”, “cognitions”), but the arch they form.

### Three-Term Contingency, Affordance, Affording of Standards

In spite of prevention in associating the term behavior with behaviorism (Sembera 2007), the notion of *operant behavior* in Skinner’s radical behaviorism is offered here as a prototype of comportment as an intentional subject-world arch (*fungierende intentionalität, intentionalité opérant, Verhaltung*), not without specifications and extensions. Within the well-known, but not emphasized strongly enough, affinity between radical behaviorism and phenomenology (Fallon 1992; Pérez-Alvarez and Sass 2008; Scharff 1999), it is important to stress an essential aspect of operant behavior: its configuration as a functional unit of three terms called the *three-term contingency*.

The terms of the contingency are behavior (typically a manipulative action or verbal act), a possible effect to occur (reinforcer) and a present situation that makes the action propitious for such effect (discriminative stimulus). The effects given reorganize the situation and following actions. The simplest notation of contingency is: S^D^: R_o_ ➔ S^R^ (S^D^ = discriminative stimulus, R_o_ = operant class, S^R^ = reinforcer), which is read: in the presence of or given stimulus (S^D^) a certain behavior (R_o_) probably has a certain effect (S^R^). Without this discriminative aspect, or in presence of another, that behavior does not work, and another may even do so. In turn, S^D^ may depend on the presence of another event (conditional discrimination). The S^D^-R_o_ relationship does not describe a linear causal relation, but *discriminative*, which might indeed be conceived as *catalyzing* one or another possibility or trajectory (Valsiner 2014a), in this case, operant behavior. The R_o_-S^R^ relationship describes a final causality (Pérez-Álvarez 2009, 2017). The final cause is not explained as much by a handy mental representation within oneself (*petitio principii*, begging the question), as the fact of one’s being *within* a configuration of sense, context or situation. Driving a vehicle offers an example of complex operant behaviors. Manipulative operations at the steering wheel, of the legs on the pedals and perceptive on the road and rearview mirrors constitute constantly changing contingencies, *catalyzed* by the successive configurations of the situations that arise. Notice the intentionality inherent in forward-oriented, operant-behavior.

The three-term contingency constitutes a unit of sense, far from its apparently linear schema, as Spanish philosopher Juan B. Fuentes has shown. The three-term contingency constitutes a functional, temporal, dynamic, gestalt unity. Discriminative aspects of a *present* situation are functionally co-related with possible *future* events by means of molded *behaviors* put into play by a subject in such a situation. The behavior is the relationship between present situations and possible co-present situations leading to new present situations and so forth (Fuentes 2011; Fuentes and Quiroga 1999). In a more general manner, psychology is revealed as a “science of the zone between the *existing* and the *possible*” (another sense of its liminal nature), “where what is observable here-and-now is oriented towards some future occurrence” (Valsiner 2014b, p. 9).

The notion of comportment introduced here with reference to operant-behavior is inscribed on the border of irreversible time, within a process continually *interdependent* on the situation, typically in a social context (Valsiner 2016). Situating comportment (human *psyche*) on the border of time between the past and the future is fundamental against the hypostasis of psychological phenomena and capture instead its irreversibility.

Beyond the letter of Skinner, the comportmental properties of the environment would have to be discussed, affording comportment of the subjects. The notion of affordance introduced by James Gibson accounts for environmental properties related to perceiver-actor. According to Gibson:

An affordance cuts across the dichotomy of subjective-objective and helps us to understand its inadequacy. It is equally a fact of the environment and a fact of behavior. It is both physical and psychical, yet neither. An affordance points both ways, to the environment and the observer. (Gibson 1979, p. 129).

Now beyond Gibson, affordances are not limited to their functional role, but may also have an aesthetic value. According to Jaan Valsiner:

The affordances of objects afford the construction of semiospheres around the objects, which would link the mundane action with cultural meaning systems far beyond the immediacy of action object. (Valsiner 2014b, p. 144).

Along this line, the notion of affordance can be extended to “affording of social standards” related to collective practices and in gerund form indicative of the dynamic process of person-environment transactions. Affording of standards refers to the point where a person meets and deals with society through a great variation of standards, which guide collective practices as well as a person’s sense-making and development (Pedersen and Bang 2016, p. 474). Affording of standards leads to the process of subjectification.

### From Subjectified Subjectivity to Objectified Subjectivity

The operant-behavior perspective of radical behaviorism (radical = total and root) finds the roots of the subjective world (“private”) in social practices: how the “verbal community” teaches individuals starting as children to account for a part of the world only observable of oneself (Skinner 1945). Thus Skinner refers to at least four ways others teach children and they learn to extend and call what they feel, for example, by the connection between private stimuli and public stimuli which produce them (others say “hurts” and call what the child feels an “ouch” when observing that he has been hit). To Skinner, there is no ontological or epistemological problem here of the “truth of correspondence”, but only the ambiguity inherent to psychological terms due to the unsystematic contingencies which constitute the “private world” (Skinner 1945).

In relation to subjectified subjectivity, an objectified subjectivity process may now be conceived consisting of “coming into contact” with the pre-verbal feelings through the handle, focusing-type words of Eugene Gendlin. More particularly, it refers to the new conception of validity of introspection in the dynamic terms of a process of becoming aware of it and describing it (Bitbol and Petitmengin 2013; Weger and Wagemann 2015a). This process consists of an enlargement of the field of attention and contact with re-enacted experience, rather than ‘looking within’ (Bitbol and Petitmengin 2013). A new consideration of unconsciousness as “dark matter of the mind” (infallible and unspoken), is opened to post-Cartesian psychoanalysis (phenomenological contextualism; Storolow 2013) and a non-nativist conception of language and the mind (Everett 2016).

In contrast to the dualist, mental/behavior (mind/world, internal/external) dichotomy, the subject-comportment duality makes it possible to understand subjectivity as an extended part of the world (not a world apart) and the world-of-life as an extension of subjectivity with its comportmental properties (affordances, affording of standards), co-evolved from a history of mutual constitution, always open and running.

## Conclusions and Discussion

It has been shown that psychology as a science of the mind and behavior suffers from a dualistic ontology consisting of the distinction between two substances, one mental, inner, unobservable, and the other behavioral, outer, observable (Schacter et al. 2015; Westerman and Steen 2007; Yela 1987). It is believed that this dualism has been overcome by adopting the scientific method from the natural sciences. However, the scientific method itself shelters an implicit dualistic metaphysics which contributes to that dualism (Packer 2011; Slife et al. 2012). The distinction between two planes of epistemological order, one hypothetic-deductive and the other empirical-observational, along with subject/object, theory/practice and value/fact dichotomies, ends up as feedback to the dualism which it has supposedly overcome (Bishop 2007; Yela 1987).

This article proposes an alternative pluralistic ontology with epistemological implications for the scientific status of psychology. Based on these two conclusions, it discusses a series of hot points in psychology concerning its scientific particularity, its mediating role with regard to the other sciences and others, as well as the proposal’s capacity for integration.

### Placing Psychology on an Ontological Map

As an alternative to rocking back and forth between dualism-monism, a pluralistic ontology is proposed which distinguishes three broad genres of materiality or realities. The genre of psychological realities or phenomena (typically subjective and behavioral events) situates it between and in relation to biophysical and supraindividual realities (cultural, abstract), without being reduced to them. According to this ontology, psychological phenomena would have a triple ontological dimension, more or less conspicuous or relevant depending on the case. The Necker’s cube offers an example of how an essentially psychological phenomenon (ambiguous perception) can have biological (neurophysiology of perception) and objective, in this case geometric-abstract (laws of polyhedrons) dimensions at the same time. Without these non-psychological dimensions, the psychological phenomenon itself would not exist, but neither is it reduced to or deduced from them.

Even when all the psychological phenomena involve a neuronal biological dimension, the neurophysiological process may be irrelevant. In the classic example of the “wink”, the neuromuscular innervation involved (undoubtedly complex) does not allow a simple blink to be distinguished from a wink. In any case, the wink is lost in the subpersonal neurophysiological explanation and on the contrary, makes sense in the context of an interpersonal relationship. Furthermore, even when certain phenomena, such as crying, have a conspicuously psychological aspect, crying may in some cases end up “falling” into a generic bodily process (Plessner 1970) or in another be more than anything else the correct way to participate in an event (wailer). In both cases, the intimate personal psychological aspect would yield to impersonal functions where in the first case, the body takes it over and in the second, it dissolves into a collective event.

### Epistemological Implications for the Scientific Status of Psychology

The location of psychological phenomena on an ontological map has implications related to the type of science psychology is. The options are reduced to two: whether psychology is a natural or a human science. However, in spite of dealing with non-natural phenomena, psychology has been institutionalized as a natural science. This is for the expediency of applying a supposed natural scientific method which cannot even be said to exist in the natural sciences. The problem of a psychology based on the supposed natural scientific method, is that, in addition to the dualism which it feeds instead of overcoming, the marginalization of a variety of traditions without that problem or “complex” of the method and the scientific credibility crisis it has arrived at (Ferguson 2015; Lilienfeld 2012).

Without the natural science label, psychology does not stop being a science, as a type of human science, perhaps more humble, but more worthy and real. As its identification as *science* is nonetheless not trivial, the problem here is not the lack of options, but their variety, such as human, cultural, social, hermeneutic, contextual or behavioral. Each of these options is both applicable to psychology and shared with a group of sciences that have the human being as their subject. Each “descriptor” has its history, aspect and tone which should be kept in mind whenever it is “chosen”. Insofar as we are concerned, in keeping with the emphasis placed on the subject’s behavior, psychology would be spoken of as a behavioral science (in the compartmental sense), focusing on the human subject (person), as a hermeneutic subject with all its subjectivity always situated within a cultural context (semiotic, normative, historic).

### Interactive, Ephemeral and Liminal Events, but with Scientific Objectivity

As behavioral science, psychology is a *peculiar* science. To begin with, it deals with interactive realities (not naturally fixed), which can be influenced by the research process itself. Objects of the behavioral sciences are themselves interactive subjects, beings if there ever were any. Moreover, psychological phenomena are ephemeral (irreversible), occurring on the boundary between past and future, “the present”. Yet the world of life is relatively stable due to its institutional, normative nature, mediated by signs (“semiosphere”). For this same reason, psychological phenomena are also sufficiently regular to establish a science, psychology, as a *liminal* science in the intersection of natural and human sciences.

Psychological phenomena, within their uniqueness, are still similar and form structures. Their similarity enables generalization, not as a statistical mean, but as the principles that “govern the emergence of ever new uniqueness” (Valsiner 2014b, p. 257). Structures, on the other hand, involve functional relationships of parts within a whole (Gestalt). The three-term contingency has been shown as a present-future gestalt unit through operant behavior (Fuentes 2011), and affordances as functional relationships of actions with daily objects and systems with cultural significance (Valsiner 2014b, p. 144).

Let us take two more examples of structures or gestalts as dynamic units. The structural-systemic notion is constitutive of higher psychological functions “to refer to understanding according to which the world is a system composed from elements or components in specific relationships at different levels of analysis.” (Toomela 2016, p. 98, note). The notion of structure or Gestalt is being claimed in psychopathology as an alternative to polythetic, criterial classification based on symptoms, as if people had loose symptoms. The “notion of gestalt entails an interplay of factors that extend beyond the subject to include not only a mental state, but also the patient’s engagements with the environment and others. For instance, detecting a delusion involves taking into account not only the patient’s verbal contents but also his experiences, way of arguing, relational style and relevant historical information” (Parnas 2015, p. 285).

### The Mediating Role of Psychology

Like a liminal science in the middle of a *tectonic fault* between the great plates of natural and human sciences, psychology is always in a critical position, in perpetual crisis, at risk of collapsing to the thread of the reductionist trend of the moment. However, it does not collapse, no matter how many reductionisms close in around it. But neither does it end up standing up and defining its mediating role between both realities and types of science. The fact that it does not collapse in spite of everything speaks more of a firm presence as an ontological entity than of a mere subsistence by inertia or interests. However, its conception as a supposedly natural science of the mind and behavior, or when applicable, as a cognitive neuroscience with its dualism-monism, does not help define its position and role on the map of an ontological and epistemological plurality.

In this respect, psychology has been conceived on the basis of a “tectonics of the person” with its ontological three-dimensionality (Ortega y Gasset 1924/1966). According to the triple ontological dimension coparticipation of human behavior is conceived in the configuration of biophysical and cultural realities themselves and their relationships. In this sense, psychological activities as constructive and transforming human actions in the world, and psychology as a science among other sciences would have a mediating role, not reducible to them, but not reducing them either. This mediating role, constructive and transforming, of psychology assumes a notion of comportment as a constitutive relationship of the subject with the world different from the notion of behavior as if it were an instrument of the mind, as well as a notion of an *embodied, embedded, and enacted* subject, practical-manipulative, different from the inner mind or in this case, the brain as information processor (Jacobs et al. 2014; Merleau-Ponty 1942/1963; Thompson 2007).

According to this emphasis on the subject and comportment, the constructive and transforming role of human action would be better understood (than in terms of mind and behavior according to the problems mentioned), not only in the “semiosphere” (society, culture, institutions, norms, ways of life), but in the “biosphere” (habitats, niches) and even in the “geosphere” (Anthropocene). Neither is culture alone a transforming agent, but does so through human actions, nor can genes be said today to be the main agents in evolution, but organisms’ behavior. Organisms’ behavior, with its potentially selective construction of niches and ways of life can alter the genome itself and end up having a mediating role in evolution (Laland and Brown 2006). In this sense, psychology would become a science mediating between behavioral (“semiosphere”) and natural (“biosphere”) sciences.

Similarly, psychology would also have a mediating role within the behavioral sciences. Thus, for example, as economists acknowledge, psychological “factors” (attitudes, fears, optimism) would be the true “actors” of economic change (inflation, deflation, bubbles). Take *irrational exuberance*, for example, according to Robert Shiller:

Irrational exuberance is the psychological basis of a speculative bubble. I define a speculative bubble as a situation in which news of price increases spurs investor enthusiasm, which spreads by psychological contagion from person to person, in the process amplifying stories that might justify the price increases and bringing in a larger and larger class of investors, who, despite doubts about the real value of an investment, are drawn to it partly through envy of others' successes and partly through a gambler's excitement. (Shiller 2015, p. 2).

Furthermore, rather than being blinded by its neuroimages, psychology sheds light on neuroscience. After all, psychology would have to come before and after neuroscientific studies: before by defining the phenomena to be studied in or by the brain and afterwards by making sense of them, because sense is not found in or deduced by the brain. The “wink” mentioned could be a starting point for studying its neurophysiology (if there were no wink as a phenomenon of interest, nobody would study it in the brain), but the correlates involved are not the wink. Its sense is still in the distal relationship with interpersonal phenomena, not in the adjacent neural proximal relationship.

### Nothing Psychological outside of Psychology

Within psychology itself, its conception as a science of the subject and comportment has thematic and methodological implications. For one thing, nothing psychological can be outside of psychology because of method, beginning with implicit prereflexive subjective aspects (unconscious, nonverbal, ineffable, “dark matter of the mind”). In this respect, the “new” conception of introspection has already been shown, not as access to a content that is there-in, according to the conception of true correspondence, but as “coming into contact” through a process of becoming aware (Bitbol and Petitmengin 2013; Weger and Wagemann 2015a).

Let us add the “new” conception of schizophrenia as an alteration of the experience itself and of the world, according to the ipseity-disturbance model (Sass 2014; see also Pérez-Álvarez et al. 2016). Beyond the psychotic symptoms, this focus reveals a characteristic alteration of basic subjectivity and way of being-in-the-world, typically prereflexive aspects, implicit, hard for the dominant positivist natural science conception to highlight. On the contrary, according to a cultural phenomenological focus, in this case, concentrating on the subject and comportment as the “intentional arch” in the above sense, essential structural aspects of schizophrenia are shown (Sass 2014). All we need to do is rethink clinical phenomena in terms of the person and his/her circumstances (Pérez-Álvarez et al. 2016), including appropriate methods, in this case semi-structured interviews exploring various aspects of subjectivity (Sass et al. 2017).

An approach to psychology such as the proposal on explicit ontological and epistemological bases can calibrate the scientific quality of the typically positivist neurocognitive behavioral approach in clinical use. Taking Attention-Deficit/Hyperactivity Disorder (ADHD) as a test bed, it can be shown how standard scientific research creates objective methods with the preconceptions which sustain the “disorder” and receives feedback from the research itself (Pérez-Álvarez 2017). As shown, the standard positivist research method itself incorporates implicit metaphysics (typically dualistic-monistic), far from its intended exempt objectivity, conforming to the data. After uncovering the blind points of the ADHD neuroscience, including its rhetoric and self-confirming research, the problem of the person who receives the “ADHD” diagnosis may be no more than a certain style of comportment, not “symptoms”. As such, the supposed best diagnosis would be understood as a “semiotic mediator” at the service of a variety of interests, not a clinical entity (Brinkmann 2014; Pérez-Álvarez 2017).

### Beyond the Dispute of the Scientific Method

The turn of a pluralistic qualitative inquiry pointed out at the beginning (Gergen et al. 2015; Valsiner et al. 2016) may be seen as a promise awaiting its institutional implementation. It is expected for theoretical inquiry and qualitative research to match the complexity of human phenomena (Gough and Lyons 2016) so “psychologists could ask any relevant research question, and use any methodology and technique that was needed in order to adequately address their research question, without much thought as to whether this was a qualitative or a quantitative approach” (Brinkmann 2015, p. 171). As Brinkmann continues, perhaps the future is one of postqualitative research.

Not because psychologists stop doing interviews, fieldwork, or other kinds of qualitative work (I certainly hope not!), but because they stop defining their research endeavors in terms of a method (Brinkmann 2015, p. 171; see also Lilienfeld et al. 2015).

Then it would be on a level with the natural sciences, which do not have the typical disputes concerning the scientific method as psychology has had up to now. As Michael Mascolo says,

A debate over whether a given discipline is or is not a science would seem to be more of a battle about status and prestige than about identifying alternative pathways to reliable knowledge. A better question might be, given its subject matter, how can we study psychological processes in systematic, reliable and useful ways? If such conditions can be satisfied, the question of whether or not disciplinary practices are scientific would be irrelevant. (Mascolo 2016, p. 553).

### The Problems of Replication, Hypotheses and Globalization Revisited

The problem of replication (Open Science Collaboration 2015) would be seen as something inherent to psychological entities due to their interactive nature and depending on the number of “variables”, not as a scientific deficit of psychology. Replication is a problem of and for the positivist scientific method which takes its criterion of truth based on its principle of verification (Loscalzo 2012). Without the arrogance of eternity, scientific truth seems more humble, but perhaps more truthful, in particular, because it respects human sciences. However, replication continues being relevant to establishing regularities and generalizations. According to Mascolo:

There is both order and variation. It is to say that if we want to study that order, we must examine what real people actually do in real time and in various times and places. Under such assessment conditions, the complexity of psychological order and variation become clearer, and traits become less trait-like. (Mascolo 2016, p. 550).

The problem of *suspicious* hypotheses (Fanelli 2010) may have to do with a double particularity of psychological knowledge. One particularity refers to a good deal of technical vocabulary of psychology forming part of ordinary language. The other refers to the interactive and hermeneutic nature of the participants in psychological studies. Human subjects in psychological experiments do not comport themselves like natural objects (Danziger 1990). In spite of all this, nothing removes the relevance of hypotheses in psychological research, more so to the extent that they are challenging, not tautological or common sense, if they really test models or theories, and if, in particular, they refute common sense (Lilienfeld 2012).

The problem of *indigenous* Western psychology being taken as universal (Christopher et al. 2014; Henrich et al. 2010) is understood according to the interactive character of human beings, susceptible to a diversity of ways of being depending on historically given and socially organized ways of life. The problem is really due to the dominant scientific attitude in psychology as a supposedly natural science which is describing how the mind of a universal subject functions. Psychologists would have to recognize “that every psychology, including U.S. psychology, is inevitably indigenous; that is, it is embedded in and a product of the surrounding culture and local societal conditions.” (Christopher et al. 2014, p. 648). According to Valsiner,

The restoration of focus on the subjective processes of culture human beings in their willful acts of living their lives is the contribution globalization can bring to Psychology.” (Valisner 2013, p. 257).

### For a more Integrative Science of Psychology

Psychology as a science of subject and of comportment further enables integration of other approaches of psychology: behavioral (functional contextualism, radical behaviorism), cognitive (embodied, embedded, enacted, amalgamated mind), ecological-cultural (affordances, affording standards, semiotics), phenomenological-existential (being-in-the-world, hermeneutics, first-person perspective); humanistic-experiential (focusing, person-centered), psychoanalytic (phenomenological contextualism, new introspection), in pursuit of a *qualitative* psychology as a science of the human *psyche* (Gergen et al. 2015; Valsiner et al. 2016). The persons’ own subjective worlds have been distanced from science, leading in our twenty-first century to renewed calls for “bringing the subjective” into the science of psychology (Valisner 2013, p. 257).
